# Scale and spread of innovation in health and social care: Insights from the evaluation of the New Care Model/Vanguard programme in England

**DOI:** 10.1177/13558196221139548

**Published:** 2023-01-11

**Authors:** Julie MacInnes, Jenny Billings, Anna Coleman, Rasa Mikelyte, Sarah Croke, Pauline Allen, Kath Checkland

**Affiliations:** 1 Centre for Health Services Studies, 2240 University of Kent, Canterbury, Kent, UK; 2 Division of Population Health, School of Health Sciences, University of Manchester, Manchester, UK; 3 London School of Hygiene & Tropical Medicine, Health Services Research Unit, London, UK

**Keywords:** scale and spread, innovation, implementation

## Abstract

**Objective:**

Little is known about how to achieve scale and spread beyond the early local adoption of an innovative health care programme. We use the New Care Model – or ‘Vanguard’ – programme in the English National Health Service to illuminate the process, assessing why only one of five Vanguard programmes was successfully scaled up.

**Methods:**

We interviewed a wide range of stakeholders involved in the Vanguard programme, including programme leads, provider organisations, and policymakers. We also consulted relevant documentation.

**Results:**

A lack of direction near the end of the Vanguard programme, a lack of ongoing resources, and limited success in providing real-time monitoring and evaluation may all have contributed to the failure to scale and spread most of the Vanguard models.

**Conclusions:**

This programme is an example of the ‘scale and spread paradox’, in which localism was a key factor influencing the successful implementation of the Vanguards but ultimately limited their scale and spread.

## Introduction

Health and social care systems are facing significant challenges due to an ageing population, increased demand for services, and limited financial resources.^1^ Creative ways of organising care through integrated services are seen as one mechanism through which these challenges can be met.^2^ As a result, transforming the way services are organized to allow large-scale integration has become a major preoccupation for policymakers.^3,4^

Commentators concur that such system transformation is required for effective, efficient, and sustainable health care.^5^ However, it has been argued that highly institutionalized and complex health care systems find this kind of transformation difficult, being slow to adapt, innovate, and improve.^6^ Change therefore happens incrementally and inconsistently, with successful innovation resembling a journey rather than a single event, characterized by processes of adoption, implementation, sustaining, spreading, and scaling up.^7^ Whilst research has explored processes of adoption and implementation, developing strong theoretical frameworks,^8^ less is known about how to achieve scale and spread beyond local early adopters.^9^ As a result, innovations have often fallen short of realising their full potential impact.^10^

Scale and spread of innovations can be conceptualized in a number of ways. Here, we define spread as ‘horizontal diffusion’ with innovations replicated in other geographical areas, commonly by informal or decentralized actions. In contrast, scaling up – or ‘vertical diffusion’ – is the deliberate and systematic approach to rolling-out a local programme more widely via meso- or national-level coordinated action. It involves building the infrastructure to support full implementation.^11,12^

A number of conceptual frameworks and models have been employed to explore the determinants of successful scaling and spreading of innovation in health care.^13,14^ Prominent amongst these is Rogers’ ‘Diffusion of Innovation’,^13^ which identifies four main elements for innovation: innovation characteristics, communication channels, time, and the social system. Greenhalgh’s^14^ seminal work introduced concepts of adoption, assimilation, diffusion and dissemination, system antecedents and readiness, and contextual factors. Building on Greenhalgh, Nolte^7^ brings together a number of factors that support implementation, scale, and spread, as shown in Table 1. We used Nolte’s conceptual framework for the current analysis.

**Table 1.** Factors supporting scale and spread of healthcare service innovation (adapted from Nolte).^7^

Factor	Key component
Organisational structure	Adaptive and flexible structures that support devolved decision-making
Leadership and management	Supportive and committed to change, including the articulation of a clear and compelling vision
Stakeholder involvement	Early and widespread, including staff and service users
Dedicated and ongoing resources	Including funding, staff, infrastructure, and time
Effective communication	Across each individual organisation and between organisations
Adaptation	To the local context and integrated with existing programmes and policies
Ongoing monitoring and feedback	Focussing on processes, occurring in a timely manner
Evaluation	Demonstrating effectiveness, including cost-effectiveness and health benefits

In this paper, we use the New Care Model (NCM) or ‘Vanguard’ programme to illustrate the processes of scaling up and spreading programmes aimed at service improvement and innovation. Costing in excess of £UK300 million,^15^ the Vanguard programme sought to increase service integration in the English National Health Service (NHS). The main organisations in the NHS infrastructure relevant to the current study are detailed in Table 2.

**Table 2.** Summary of relevant NHS infrastructure (adapted from The Kings Fund).^30^

Structure	Summary description
NHS England and NHS Improvement	National-level organisations, responsible for the quality, financial, and operational performance of all NHS organisations
System Transformation Partnerships	Operate at a regional level and bring together NHS providers and commissioners, local authorities, and other local partners to plan services to meet the needs of local communities. They cover populations of 1–3 million people
Integrated Care Systems	Operate at a regional level and since 2021 now cover the whole of England. They evolved from the System Transformation Partnerships and are a closer collaboration in which organisations take on greater responsibility for managing local resources and improving health and care for their populations
Primary Care Networks	These bring together general practices to work at scale with other local providers from community services, social care, and the voluntary sector. They cover populations of 30–50,000 people

The government’s *Five Year Forward View*^16^ described a vision for the NHS, focussed upon new ways of working, aiming to break down barriers between organisations and care sectors. It was proposed that a number of ‘Vanguard’ sites would be established to design, test, and deliver a variety of scalable and replicable NCMs, with the expectation that success would be repeated elsewhere.^17^

Five types of NCM were proposed: Primary and Acute Care Systems (PACS), Multispeciality Community Providers (MCPs), Urgent and Emergency Care (UEC), Acute Care Collaboratives (ACCs), and Enhanced Health in Care Homes (EHCH). To support the implementation of and learning from the Vanguards, an extensive support programme was established, led by NHS England. This included local evaluations of each Vanguard and a national evaluation, of which this study forms part.^18,19^

Ultimately, only one Vanguard model was scaled and spread – EHCH. The NHS’s *Long-Term Plan*^20^ set out detailed and specific support to be delivered to all care home residents by 2023/24. An additional contract, known as a Directed Enhanced Service, was introduced to offer payments to general practices if they worked together to offer these additional services.^21,22^ Services specified included a named clinical lead, a weekly ‘home round’, needs assessments and care plans, and mediation reviews. This policy is a direct legacy of the EHCH Vanguards. The NHS *Long-Term Plan* noted that ‘Enhanced Health in Care Homes (EHCH) Vanguards have shown how to improve services and outcomes for people living in care homes … with the EHCH model rolled out across the whole country over the coming decade’.^20 (pp15-16)^

Vanguards are a useful vehicle to examine here because it was the explicit policy intention that the Vanguard sites would be pilots, identifying new approaches to integrated care that would subsequently be spread more widely.^16^ Indeed, one of the initial goals of the programme was to ‘identify the most promising models that can be spread elsewhere’.^17(p20)^ However, although all Vanguard models were intended to be rolled out, only one became enshrined in national policy. This raises the question as to why one model became national policy whilst the others did not. We answer this question by examining the experiences of the Vanguards, using Nolte’s^7^ framework.

## Methods

This study forms part of the National Evaluation of the NCM, Vanguard, programme (2017–2021), funded by the NIHR Policy Research Programme. In phase 1, we reported on the design and impact of the Vanguard programme at a national level.^18^ In phase 2, we examined Vanguard experiences through in-depth case studies,^19^ and in phase 3 we examined the roll-out of the EHCH policy, including revisiting the phase 2 case study sites. This paper draws on all three phases:

***Phase 1: National perspective.*** We carried out 29 national-level interviews with current and past employees of NHS England (the NCM team, care model leads, and support stream leads), strategic account managers, advisors to the programme (national oversight group, Improvement Analytics Unit), and people involved with arm’s length bodies (regulators). We also interviewed 11 local evaluation leads and sourced publicly available documents on the programme. The interviews and document collection took place between October 2017 and March 2018.

***Phase 2: Vanguard case studies.*** We selected six case sites – two MCPs, two PACS, and two EHCH Vanguards – for the analysis. For the MCPs and PACS, we took a pragmatic approach to site selection, recruiting sites to ensure geographical spread and representing Vanguards of contrasting size and experiences. A total of 80 respondents took part in individual interviews or focus groups, representing local service commissioners (Clinical Commissioning Groups), System Transformation Partnership leads, provider organisations, local authorities, voluntary sector organisations, Vanguard programme leads, front-line staff, and patient/public contributors. It should be noted, however, that few respondents represented social care organisations or front-line care staff. We also sourced publicly available documents on each Vanguard case site. The interviews, focus groups, and document collection took place between October 2018 and July 2019.

UEC and ACCs were excluded from this analysis as UEC was never fully embedded into the programme and was only formally part of the programme for one year. The UEC model was a set of interventions that had already been established. As such, it was not experimental in the same way as the other models and therefore did not fall within the remit of our study, which focused upon evaluating the bottom-up development of new population-based models of integrated care. Similarly, ACCs were separate from others in the NCM programme. The ACCs Vanguards had specific support requirements and were less focused on whole population health care design. They were led by NHS Improvement and were not part of the national support programme.

***Phase 3: EHCH roll-out.*** We interviewed three national-level policymakers and revisited the same six Vanguard case sites in phase 2 where we interviewed 29 individuals. We also collected policy and process documentation via websites or through the respondents. Finally, we linked with a parallel project on Primary Care Networks^23^ in which 31 interviews with network staff were carried out. The interviews and document collection took place between July and December 2020.

Interviews and focus groups were transcribed verbatim and analysed using NViVO software. The analytical approach was thematic, incorporating a priori themes developed from relevant literature, alongside issues derived from the data. To preserve anonymity, unique identification codes are used. For phase 1, the phase (P) and respondent (R) only are given (e.g. P1R11). For phases 2 and 3, the phase (P), case site (S), and respondent (R) are given (e.g. P2S6R05).

## Results

This section explores the factors influencing the limited scale and spread of the four other Vanguards, alongside the EHCH roll-out. Our findings are summarized in Table 3.

**Table 3.** Summary of differences between the four other Vanguards and the EHCH.

Factor	Four other Vanguards	EHCH policy
Organisational structure	No defined organisational structure or mechanisms to enable scale and spread	The organisational structure by which the EHCH was to be implemented was specified, namely, Primary Care Networks
Leadership and management	No formal leadership or management devoted to scaling up and spreading	Roll-out was the clear responsibility of Primary Care Networks
Stakeholder involvement	Stakeholder engagement occurred	Stakeholder engagement occurred
Dedicated and ongoing resources	Dedicated resources, but little or no resources directed at scale and spread	Dedicated resources, but significantly less than was available for the other Vanguards. Often needed local top-up
Effective communication	An important factor for scale and spread, but was ad hoc and reliant on key individuals	Effective communication between care homes and other agencies was important and occurred, especially during the pandemic
Adaptation	Initiatives were difficult to codify as they were adapted to their local context from the start and so were highly context-dependent	Was easily codified into a specific framework for national roll-out. However, there was little or no adaptation to the local contexts
Ongoing monitoring and feedback	There was ongoing feedback and monitoring during implementation, but it was limited in its effectiveness	There was ongoing feedback and monitoring during implementation, which appeared more effective than with the other Vanguards
Evaluation	Local and national evaluations were part of the programmes, but evaluation was mostly aspirational	Limited robust evaluation to date

### Organisational structure

There was no defined organisational structure or mechanisms to enable scale and spread of the Vanguards. As one service commissioner said: ‘Within the Vanguard we were like our own little bubble’. (P3S1R03)

Subsequent to the Vanguard programme, integration activity in the English NHS focused upon new regional collaborations, covering large populations, known as Integrated Care Systems.^19^ We found limited evidence of any explicit impact of Vanguard activity on integration across these wider regional structures, largely because there were few mechanisms by which activity at local level in Vanguards and a developing system architecture at regional level could be linked. System Transformation Partnership leads indicated that some but not all Vanguard models would be integrated into the partnerships going forward.

In particular, developing Integrated Care Systems were not required to engage with their local Vanguards, and despite an extensive evaluation programme^24^ there were no national mechanisms by which learning from the Vanguard programme could be synthesized and incorporated into Integrated Care System plans.^19^ Where there was evidence of an explicit link between these plans and previous Vanguard activity, this was generally mediated by serendipitous factors such as the employment of a particular individual with Vanguard experience in an Integrated Care System role.

In terms of the EHCH roll-out, all areas were required to develop Primary Care Networks (through which the EHCH was to be rolled out) alongside or within existing system architecture. The arrival of COVID-19 in early 2020 accelerated the process, and in many cases Clinical Commissioning Groups stepped in to help the initial implementation, due to the lack of maturity of the Primary Care Networks. There was thus an established architecture by which EHCH services could be implemented, and these demonstrated local adaptability.

### Leadership and management

The wider Vanguard programme and the EHCH roll-out differed in that there was no formal leadership or management devoted to scaling up and spreading the other Vanguard initiatives, whilst the EHCH roll-out was the clear responsibility of Primary Care Networks, as a local system leader said:

The key leader … was chair of the Vanguard board at the time and is now chair of the Integrated Care System. So he’s added that continuity and is absolutely key to taking a number of what we’re trying to do forward, and is really passionate about ensuring that we do act as an integrated care system with all the learning from the Vanguard. (P2S5R08)

Leadership at a national level was also lacking, in that the national guidance or frameworks envisaged by NHS England were not ultimately produced. This lack of national direction at the end of the Vanguard programme and the failure to produce any ‘blueprints’ for scale and spread arguably limited their potential.

By contrast, the EHCH Directed Enhanced Service contract specified the mechanisms by which scale and spread were to be achieved. Leadership was assigned to Primary Care Networks operating at neighbourhood level, with support and co-ordination provided by the Clinical Commissioning Groups. EHCH roll-out is occurring alongside shifting structures and within a complicated web of engaged organisations, but at least a series of leaders of change were designated, as this service commissioner said:

We’re working with Primary Care Networks through their clinical leadership network. We’re working with boroughs through the enhanced health in care homes programme approach, with the managerial, operational leads, and we’re working at an Integrated Care System level on the strategic initiatives to improve our relationships with care homes. (P3S3R01)

### Stakeholder involvement

Stakeholder engagement was crucial both for the Vanguard programme and the EHCH roll-out and in both cases took place. We found little difference between them in this regard. A history of collaborative working, often over long periods, was a facilitating factor for the Vanguards and the EHCH roll-out.

For the Vanguards, many of the teams were already working together and being part of a Vanguard accelerated this, as this Vanguard team member said:

Ultimately, I think it’s how you deliver sustainable change long-term, and if you look at those parts of the country that are better at doing this … they’ve got a history of collaboration that goes back 25 years … and I think it takes time and cultural change and mutual respect and trust to be able to sustain that. (P2S2R012)

An important legacy of the Vanguards for the EHCH roll-out appeared to be the development of trusting relationships across organisations and the continuity of those relationships, as this local system leader said:

I think the thing about the Vanguard legacy was really about relationships. So [Vanguard] had all the right staff and it had really good relationships. So it had prioritised the, you know, investment in the right kind of support for care homes. And we had really, really good relationships, which helped enormously … I don’t think the organisations matter at all in lots of ways. It’s all about the continuity of the individuals. (P3S3R01)

The experience of being a Vanguard also created a desire and aptitude for system transformation, as this service commissioner said:

It definitely gives us more of an appetite, I think the system has got that can-do attitude. We’re used to doing transformation quickly and being quite innovative. I suppose for me the fact we’ve been through that journey just makes you think a little bit more about being a bit more savvy about how you maintain that longer term. (P3S5R03)

### Dedicated and ongoing resources

Both the Vanguard programme and the EHCH roll-out had dedicated resources. But there were differences in terms of the perceived adequacy of this funding and the purpose for which it was used.

Substantial funding was an important influence on the implementation of the Vanguard pilots.^25^ However, although funding was effectively ring-fenced around Vanguards, no further resources were directed at scaling up or spreading initiatives. Whilst initial Vanguard ‘value propositions’ postulated future savings which would support ongoing activity once initial funding was withdrawn,^17^ in practice this did not materialize, leaving sites to find other sources of funding if they wished to continue or spread new services or ways of working. This meant that many Vanguard initiatives were terminated^19^ impacting future initiatives as highlighted by this service commissioner:

When you do a pilot and they pull it, that dampens people’s enthusiasm for the next shiny new thing. So the GPs [General Practitioners] that were involved then [said]: ‘Why should I put in my time and effort?’ Whenever I tried to introduce something else after that [GPs said]: ‘How long will this funding last for, how much do we commit to this, is it worth doing it?’ (P3S5R04)

Additional funding to care homes at the start of the COVID-19 pandemic was important in facilitating the rapid changes required. However, this funding was considerably less than had been available for the Vanguards, as this service commissioner said:

The actual work of the Enhanced Health in Care ... Obviously, it suffered from the reduction in the sort of seed funding that the Vanguard provided, and it was difficult then to mainstream in the context of austerity as well. (P3S4R02)

The Directed Enhanced Service funding approach encouraged Primary Care Networks to implement EHCH initiatives. This funding was available to support the recruitment of new, additional staff to deliver services. However, some respondents did not think the additional funding was sufficient to deliver all elements of the service specification. As a result, additional funding was provided by some Clinical Commissioning Groups to further incentivize engagement, as this GP explained:

The CCG [Clinical Commissioning Group] are topping up, they’ve tiered the homes … So they’re doing top-up for their high-risk nursing homes … I suppose it was a bit of a sweetener. (P3S1R03)

### Effective communication

Effective communication was an important facilitator of scale and spread. For the Vanguards, this was rather ad hoc and reliant on key individuals. For the EHCH roll-out, occurring during the COVID-19 pandemic, effective communication between care homes and other agencies became a necessity.

For the Vanguards, communication and information sharing to other, often neighbouring, areas enabled a degree of spread. This was dependent on the actions of highly skilled individuals who championed the successes of the Vanguards, as this clinician in a Vanguard team member said:

I’ve had some really good feedback from clinicians in my role across different localities, and sharing - you know, so sharing ideas, and sharing data, and sharing information, and just saying, ‘These are my contact details if you do ever have anything established and want some support’. (P2S1R07)

In terms of scale, again the movement of individuals with Vanguard roles to other organisations such as the System Transformation Partnerships and Integrated Care Systems was key, as this System Transformation Partnership manager said:

When I knew I was coming to the STP [System Transformation Partnership], I asked if I could bring all that we’d built up in the Vanguard, which was a lot, over to the STP so that the ceiling became the floor for the STP. (P2S1R03)

For some, communicating and sharing information with other Vanguards was seen as a part of the responsibility of being a Vanguard. NHS England facilitated this through ‘account managers’ working across Vanguards who organized webinars and other events. In addition, inter-Vanguard visits were encouraged. Despite this cross-fertilisation, there are relatively few examples where initiatives in one Vanguard were picked up and implemented elsewhere. The notable exception to this was an initiative known as the ‘red bag’, developed by one EHCH Vanguard and implemented widely (see Adaptation).

For the EHCH roll-out, the pandemic heightened the need for clear communication and saw the development of daily bulletins both locally and between national and local levels. This was to ensure accurate understanding of the evolving situation, allow clear planning, and give unambiguous and timely guidance, as this service commissioner explained:

At one point [information] was almost changing daily, and that’s when we realised, you know, we can’t keep sending out all these emails to care homes that were frazzled anyway, staff that were very, very worried and we were confusing them. (P3S1R01)

### Adaptation

The innovation processes for both the Vanguards and the EHCH roll-out required adaptation to local contexts.^14^ The EHCH Vanguard model encouraged health services and the care home sector to develop ‘new shared models of in-reach support’.^17(p24).^ A ‘framework’ for EHCH^21^ detailed specific actions to be undertaken, including a named GP, medication reviews, and hydration and nutritional support. Such codification was also attempted for two of the other Vanguard types, MCPs and PACS. However, the MCPs and PACS Vanguards represented a diffuse and highly varied range of initiatives, and the published frameworks were non-specific and process-dominated.^18^

Arguably, the wider Vanguard initiatives were adapted to their local context from the start, in that the MCPs and PACS Vanguards were a disparate collection of a wide range of initiatives that were highly context-dependent and thus difficult to codify, as this Vanguard lead said:

When we talk about scaling or spreading, we’re talking about the scaling or spreading of a thing or a model. And my suspicion … that the thing that made the Vanguards successful to the extent that they were, was localism, and is not the adoption of a model that’s been codified nationally. (P3S2R01)

In contrast, the EHCH Vanguards were more easily codified into a specific framework.^21^ We found evidence that some EHCH initiatives had spread ‘on the ground’ before national policy caught up. But such codification was not always successful, due to lack of adaptation to the local context. The Hospital Transfer Pathway, which quickly became labelled as the ‘Red Bag’, was seen as a great success by the Vanguard that developed the initiative. Every care home resident attending hospital had a red bag containing their personal information documents, medications, and belongings. This bag accompanied them from the care home through all hospital departments. Developed in one Vanguard, the ‘Red Bag’ was adopted across the country after the end of the Vanguard programme. It seems likely that the reason the initiative was spread, unlike most other Vanguard initiatives, was due to its simplicity and the fact that it had a readily identifiable symbol – the red bag itself. However, the ‘Red Bag’ idea failed to be adapted to the context of the new settings, which had different service configurations and levels of organisational engagement, for example, and therefore met with more limited success than that claimed by the original Vanguard. As this service commissioner explained:

The Red Bag, for us, just didn’t work … and we spent a fortune on all those Red Bags, and so much time and effort. But it’s the other variables, isn’t it? It’s the fact that actually we’ve got a massive Acute [Hospital] Trust that just wasn’t on board. You know, we’ve got an ambulance service that covers five counties, that, you know, there’s a whole load of reasons why. (P3S5R05)

### Ongoing monitoring and feedback

Ongoing feedback and monitoring during implementation were features of both the Vanguard programme and the EHCH roll-out. However, the impact of such activities appeared to be limited, especially for the Vanguards. Here, local evaluation teams were contracted to deliver quasi-scientific correlations and testing of causal assumptions, to establish the effectiveness of the interventions in a real-world context. Although the local evaluation teams fed back interim findings, this was often either too late for Vanguards to change course or there was a desire, and associated pressure, to demonstrate success against nationally determined parameters.^25^

This was reflected in tension between some of the Vanguards and their evaluators, where the Vanguards saw the evaluators’ role as confirming success rather than using the feedback as a mechanism to learn and refine their activities, as this local evaluator said: ‘They thought they were paying us to give us positive results but in order to maintain our integrity we had to be as neutral as possible’. (P1R11)

Increasingly, the Vanguards came under pressure to demonstrate success against nationally determined outcome measures that did not necessarily align with local objectives.^18^ The speed at which they were expected to demonstrate results added to this pressure. As a result, there was a sense that the rich, contextual learning around the mechanisms for implementation that took place within the Vanguards was somewhat lost, especially at a national level. This is evident in the EHCH roll-out, in that whilst service changes have been specified in the EHCH Directed Enhanced Service contracts, the process and mechanisms for implementation have not. There was some evidence that the EHCH roll-out during a global pandemic necessitated continuous reflection and changes in direction in response to the rapidly changing situation. As this service commissioner said:

Although Covid’s been a really bad time, for us as a team, it’s really given us time to just step back a little, and look deeper into issues, and highlight them. (P3S2R03)

However, whilst EHCH teams could reflect on what was going on within each PCN, there was little evidence of learning across the wider PCN network.

### Evaluation

Formal evaluation was a built-in feature of the Vanguards through local and national evaluations at the end of the programme. To date, there does not seem to be a national plan for evaluating the EHCH roll-out. For the Vanguards, it was expected that local evaluations would complement national interrogation of outcome metrics by examining each Vanguard’s activities in depth.^16^ It was anticipated that local evaluations would capture and evaluate the transformative changes delivered by the Vanguards and explain how, and in what context, the changes have occurred. The intention was to share learning both between the Vanguards and more widely in order to promote replicability and scale-up and embed a culture of evaluation and knowledge sharing.

The Vanguards made a number of claims to success, although often these were aspirational. They included the formation of new collaborations and opportunities to work across traditional boundaries, changing culture, building strong relationships, more appropriate referrals, better use of resources, and quality of care, for example, as identified by this Vanguard team member:

The multidisciplinary teams were definitely a success. You should see them, they're quite heart-warming to see. All those different individuals around the table, really concentrating on the person in the centre of the care and actually trying to work out what's best and what will meet that person's wishes and goals and needs. (P2S1R03)

Currently, largely as a result of the rapid EHCH roll-out, there is limited robust evaluation at the local level. However, there were examples where services were being re-configured as a result of evaluation. As with the Vanguards, claims to success are emerging, as this Care Home Team lead said:

Within the sort of first six months, we actually had some excellent outcomes from this team, with reduced admissions into hospital. So that gave the opportunity then, to go back to our Commissioning and Policy Development Committee to say look, you know, ‘We need to substantiate and embed this team on a permanent basis’. (P3S2R02)

## Discussion

Our study suggests that the general scaling up of the Vanguard programme was difficult to achieve. We found little evidence that the service changes introduced locally had any significant influence on subsequent service developments. Lack of leadership or direction near the end of the Vanguard programme, lack of ongoing resources, and limited success in providing real-time monitoring and evaluation may all have contributed to the failure to straightforwardly scale and spread most of the Vanguard models.

However, EHCH – designed to improve care in care homes – was subsequently scaled up and spread via a national contractual programme, mandating the delivery of specific services to all care homes in England. The EHCH model was the easiest to codify into a model of care, in part because the target client group was small and relatively homogeneous, and because services in this area were generally undeveloped. Existing outcomes were relatively poor,^26^ making improvement easier to demonstrate. It is clear that national leadership and the provision of ongoing resources were probably the key differences between the EHCH model and the other Vanguard types. However, at the same time, we found that the very codification that made it possible for the model to be scaled up and spread via a national contract made implementation more difficult, as local areas wrestled with the difficulties associated with adapting the prescribed model to their local circumstances. When specific initiatives were straightforwardly spread (e.g. the ‘red bag’), the lack of contextual ‘fit’ meant that it often had little impact. Finally, the limited resources provided for the EHCH roll-out (compared with those available for the Vanguards) impacted upon the ability to make fundamental changes to services.

We are therefore left with a ‘scale and spread’ paradox. The Vanguard programme as a whole was predicated on the idea that promising new ways of providing services would be prototyped and codified, allowing others to easily adopt them, but the very factors that supported successful local implementation of the prototypes – a permissive approach allowing local innovation, the provision of significant resources, and strong, long-term trusting relationships – themselves mitigated against successful top-down scaling and spreading, where such factors are unlikely to be present.

These findings support Nolte’s conclusions, highlighting the importance of allowing local areas to adapt innovations to fit their local circumstances.^7^ There is also resonance with the ‘replicability problem’ articulated by Horton et al.,^27^ who described the challenge of replicating new interventions. Despite mechanisms to encourage uptake, these authors state that the last 70 years of NHS history have shown that mandating action does not automatically bring about the desired change. Teams on the ground must be able to adapt and implement a new intervention to enable it to work in their own setting. Staff may need new skills or learn new techniques. There may also be a need for culture change, relationship building, and new ways of working or undoing entrenched habits.

This leaves policymakers with a significant challenge: if the approach taken in the Vanguard programme is unlikely to succeed, how should widespread beneficial service change in health systems be engineered? Our study provides some hints as to the factors which might be helpful. Health system management that supports ongoing, local collaborative activity is important. If such activity becomes the norm, when significant service changes need to be made (supported by top-down incentives and leadership), the required trust will be present, fostered by long-term engagement of individuals over time. This, in turn, suggests that investment in relationship building between organisations and the provision of high-quality human resource management to encourage long-term staff retention will pay dividends. Moreover, it suggests that the restless reorganisation of health systems prevalent in the UK and beyond is probably unhelpful, with repeated change resulting in ‘disorganisation’.^28^ Adequate resource provision is also clearly important, and evidence from economics suggests that appropriate incentivisation, alongside clarity around desired outcomes, supports implementation and can facilitate improved performance.^29^ For the EHCH policy, this suggests that a more helpful approach might have been to clearly identify desired outcomes, such as reductions in admissions to hospital or improvements in clients’ experiences, but to leave the mechanisms by which these are achieved to be locally determined, perhaps supported by a menu of potential approaches.

## Limitations

There are two main limitations with this study. First, there is the confounding factor of COVID-19. The implementation of the EHCH policy took place during the pandemic. This accelerated some aspects of the roll-out, but prevented a more careful or staged implementation, with local areas implementing what they could as fast as possible. While we acknowledge the considerable impact of the pandemic on care homes and primary care, a detailed discussion of its impact is beyond the scope of this paper.

Second, there is the lack of data around the implementation of the Vanguard programme from a social care perspective. Although social care organisations were part of the Vanguard initiatives in some cases, the dominant focus was on health services delivery, with Vanguard led by health care organisations or clinical commissioners.

## Conclusion

Our study has argued that the opportunity to learn from the Vanguard programme has not been systematically pursued. There is limited evidence that the subsequent System Transformation Partnership and Integrated Care System programmes built upon Vanguard experiences were comprehensively evaluated.

There is an opportunity to use such evaluation to develop more detailed guidance as to how policy innovation at the local level might best be structured and managed. We consider Nolte’s framework a useful lens through which to examine the processes of scale and spread of health care innovations.

## References

[1] Lloyd-Sherlock P Billings J Giacomin K, et al. Meeting the complex challenge of health and social care provision for rapidly-ageing populations. Introducing the concept of ‘avoidable displacement from home. Rep Public Health 2020; 36: 1–12. 10.1590/0102-311X00162819 32267376

[2] Raus K Mortier E Eeckloo K. Challenges in turning a great idea into great health policy: the case of integrated care. BMC Health Serv Res 2020; 20: 130. 32085770 10.1186/s12913-020-4950-z PMC7035709

[3] Hunter DJ Erskine J Small A, et al. Doing transformational change in the English NHS in the context of ‘big bang’ redisorganisation. J Health Organisation Management 2015; 29: 10–24. 10.1108/JHOM-01-2014-0019 25735550

[4] Maniatopoulos G Hunter DJ Erskine J, et al. Implementing the new care models in the NHS. J Health Organ Management 2019; 34: 325–344.

[5] Wutzke S Benton M Verma R. Towards the implementation of large scale innovations in complex health care systems: views of managers and frontline personnel. BMC Res Notes 2016; 9: 327. 27352864 10.1186/s13104-016-2133-0 PMC4924288

[6] Cote-Boileau E Denis J Callery B, et al. The unpredictable journeys of spreading, sustaining and scaling healthcare innovations: A scoping review. Health Res Policy Syst 2019; 17: 84. 31519185 10.1186/s12961-019-0482-6 PMC6744644

[7] Nolte E. How do we ensure that innovation in health service delivery and organization is implemented, sustained and spread? Policy Brief, Health Systems for Prosperity and Solidarity, World Health Organization. https://www.euro.who.int/__data/assets/pdf_file/0004/380731/pb-tallinn-03-eng.pdf (2018, accessed 19 March 2021).

[8] May C. Agency and implementation: understanding the embedding of healthcare innovations in practice. Soc Sci Med 2013; 78. 10.1016/j.socscimed.2012.11.021 23246396

[9] Hemmings N Hutchings R Castle-Clarke S, et al. Achieving scale and spread. Learning for innovators and policy-makers. https://www.nuffieldtrust.org.uk/research/achieving-scale-and-spread-learning-for-innovators-and-policy-makers (2020, accessed 20 Oct 2021).

[10] Willis CD Riley BL Stockton L, et al. Scaling up complex interventions: insights from a realist synthesis. Health Res Policy Syst 2016; 14: 88. 27993138 10.1186/s12961-016-0158-4 PMC5168709

[11] Greenhalgh T Papoutsi C. Spreading and scaling up innovation and improvement. BMJ 2019; 365: l2068. 31076440 10.1136/bmj.l2068 PMC6519511

[12] Eaton J McCay L Semrau M, et al. Scale up of services for mental health in low-income and middle-income countries. Lancet 2011; 378: 1592–1603. 22008429 10.1016/S0140-6736(11)60891-X

[13] Rogers EM. Diffusion of Innovations*.* New York: Free Press, 2019.

[14] Greenhalgh T Robert G MacFarlane F, et al. Diffusion of innovation in service organisations: Systematic review and recommendations. The Millbank Q 2004; 82: 581–629. 10.1111/j.0887-378X.2004.00325.x PMC2690184 15595944

[15] National Audit Office. Developing new care models through NHS Vanguards. Developing new care models through NHS vanguards. https://www.nao.org.uk/wp-content/uploads/2018/06/Developing-new-care-models-through-NHS-Vanguards.pdf (2018, accessed 20 Oct 2021).

[16] NHS England. Five year forward view. https://www.england.nhs.uk/wp-content/uploads/2014/10/5yfv-web.pdf (2014, accessed 17 April 2021).

[17] NHS England. The forward view into action: New Care Models: update and initial support. https://www.england.nhs.uk/wp-content/uploads/2015/12/acc-uec-support-package.pdf (2015, accessed 17 April 2021).

[18] Checkland K Coleman A Billings J, et al. National evaluation of the Vanguard new care models programme: Interim report: Understanding the national support programme. https://www.research.manchester.ac.uk/portal/en/projects/national-evaluation-of-the-vanguard-new-care-models-programme(1444648f-0543-4162-ac50-83e05845738c)/publications.html (2019, accessed 9 May 2021).

[19] Checkland K Coleman A Croke S, et al. National evaluation of the Vanguard new care models programme report of qualitative case studies: Understanding system change. https://prucomm.ac.uk/august-2021-national-evaluation-of-the-vanguard-new-care-models-programme-report-of-qualitative-case-studies.html (2021, accessed 14 June 2021).

[20] NHS England. The NHS long term plan. https://www.longtermplan.nhs.uk/wp-content/uploads/2019/08/nhs-long-term-plan-version-1.2.pdf (2019, accessed 17 April 2021).

[21] NHS England. The framework for the enhanced health in care homes. https://www.england.nhs.uk/publication/the-framework-for-enhanced-health-in-care-homes/ (2016, accessed 17 April 2021).

[22] NHS England and NHS Improvement. The framework for enhanced health in care homes, version 2. https://www.england.nhs.uk/wp-content/uploads/2020/03/the-framework-for-enhanced-health-in-care-homes-v2-0.pdf (2020, accessed 17 April 2021).

[23] Hammond J Checkland K Warwick-Giles L, et al. Understanding the development of Primary Care Networks: interim report, Policy Research Unit in the Health and Care System and Commissioning (PRUComm) https://prucomm.ac.uk (2020, accessed 17 March 2021).

[24] NHS England. Evaluation strategy for the new care model vanguards. https://www.england.nhs.uk/wp-content/uploads/2015/07/ncm-evaluation-strategy-may-2016.pdf (2016, accessed 17 April 2021).

[25] Wilson P Billings J MacInnes J, et al. Investigating the nature and quality of locally commissioned evaluations of the NHS Vanguard programme: an evidence synthesis. Health Research Policy Systems 2021; 19: 1–10. 33845858 10.1186/s12961-021-00711-3 PMC8042862

[26] Morciano M Checkland K Billings J, et al. New integrated care models in England associated with small reduction in hospital admissions in longer-term: A difference-in-differences analysis. Health Policy 2020; 124: 826–833. 32595094 10.1016/j.healthpol.2020.06.004 PMC7386936

[27] Horton T Illingworth J Warburton W. The spread challenge: How to support the successful uptake of innovations and improvements in health care. The Health Foundation*.* https://www.health.org.uk/publications/the-spread-challenge (2018, accessed 20 Oct 2021).

[28] Smith J Walshe K Hunter DJ. The ‘redisorganisation’ of the NHS. BMJ 2001; 323: 1262–1263. 11731374 10.1136/bmj.323.7324.1262 PMC1121736

[29] Exworthy M Frosini F Jones L, et al. Decentralisation and performance: Autonomy and incentives in local health economies. Technical Report. NCCSDO, Southampton. https://researchonline.lshtm.ac.uk/id/eprint/18627 (2010, accessed 7 May 2021).

[30] The Kings Fund. How is the NHS structured? Available at: https://www.kingsfund.org.uk/audio-video/how-is-nhs-structured-funding-flow (2020, Accessed 14 Jul 2022).

